# Remarks on the Lac Sulphuris

**Published:** 1804-05-01

**Authors:** 

**Affiliations:** Cassell


REMARKS ON THE LAC SULPHURIS, *by* Dr. PIEPENBRING, *of Cassell.*

THE preparation of sulphur, known under the name of lac sulphuris, or sulphur præcipitatum, has been considered in almost all modern pharmacopœias and works on materia medica, as not being different in quality from pure sulphur; to which it is said to be only preferred in unguents guents on account of its white colour. According to my observations, however, there seems to be a material difference in the chemical nature, as well as in the mode of operating on the living body, between this preparation and pure sulphur. Its extremely white colour seems to indicate, that by the previous solution and precipitation of sulphur something is either added to it or it has been deprived by that process of a certain substance. If the opinion of M. Van Mons be well grounded, who considers sulphur as an oxydated substance, we might be led to think, that the colour of lac sulphuris originates in a loss of oxygen; but this idea being not sufficiently confirmed, it seems rather probable, that it has received something to which the white colour is to be ascribed; I therefore adopt the following process in preparing precipitated sulphur. At the moment the sulphurated kali is infused with water, and the solution preparing to be made, part of the water is decomposed, and the oxygen, which is thus disengaged, adheres to a part of the sulphur, forming with it oxydated sulphur, while the hydrogen of the water combines itself with another part of the sulphur, thus forming sulphurated hydrogen; the whole mixture is in this manner changed to hydrogenated sulphurated kali, which consists of kali, oxydated sulphur, sulphurated hydrogen, and water; the caloric of the decomposed water being received by the sulphurated hydrogen, forms sulphurated hydrogen gas. On adding to this mixture any acid, viz. sulphuric acid, it is united with the kali, while the oxydated sulphur is separated, falling to the bottom in form of a white powder. The proximate constituents of this preparation are consequently oxydated sulphur, oxydated sulphurated hydrogen; and the remote constituents, sulphur, oxygen, and hydrogen. But whatever its chemical nature may be, it certainly seems to operate in a different manner on the animal economy; its action is quicker and more efficacious than that of pure sulphur; it is more easily digested in the stomach, and being less heating than common sulphur, it may be given in larger doses; it particularly promotes the alvine excretion, and being less disagreeable than sulphur, it may be continued for a longer time.

The best method of preparing lac sulphuris is the following. Take of pulverised sulphur two pounds, mix it with four pounds of pulverized pure kali, until they unite into an uniform powder. After being thoroughly dried, put it into an iron vessel, which has been previously heated on a coal fire. The vessel must be well shut. As soon as as the mass begins to melt, stir it occasionally with an iron spatula till a portion of it, when taken out, is soluble in hot water, which is a sure criterion of the process being finished. Take the vessel from the fire and let it cool; then pour eight pounds of water into it, stirring the mixture till the whole be dissolved; and when it is cold, pour it into a glass vessel and add two more pounds of water. In this vessel it must stand for two or three days, till it has obtained a yellowish colour. The solution of hydrogenated sulphurated kali is poured into another vessel, and the remaining sediment put on a piece of thick white linen, which has been previously moistened, and about 4 pounds of water gradually run over it. Meanwhile, mix 6 pounds of water with 2 pounds of white concentrated sulphuric acid, and let the mixture cool. Having added the water which runs over the sediment to the rest of the solution, gradually infuse into the whole mixture small portions of the diluted sulphuric acid, which must be continued as long as any effervescence is perceived. When the sulphur is in this manner precipitated, the liquor is suffered to run off and the residuum is properly edulcorated. The quantity obtained from the whole mass will be about 18 ounces; it will appear of a beautiful white colour.

